# Drivers of temporal beta diversity of a benthic community in a seasonally hypoxic fjord

**DOI:** 10.1098/rsos.172284

**Published:** 2018-04-18

**Authors:** Jackson W. F. Chu, Curtis Curkan, Verena Tunnicliffe

**Affiliations:** 1 Department of Biology, University of Victoria, PO Box 3080, Victoria, BC V8 W 2Y2, Canada; 2 School of Earth & Ocean Sciences, University of Victoria, PO Box 3080, Victoria, BC V8 W 2Y2, Canada; 3 Fisheries and Oceans Canada, Institute of Ocean Sciences, Sidney, BC V8 L 4B2, Canada

**Keywords:** oxygen, ecological time series, time-lapse camera, Saanich Inlet, beta diversity, Salish Sea

## Abstract

Global expansion of oxygen-deficient (hypoxic) waters will have detrimental effects on marine life in the Northeast Pacific Ocean (NEP) where some of the largest proportional losses in aerobic habitat are predicted to occur. However, few *in situ* studies have accounted for the high environmental variability in this region while including natural community-assembly dynamics. Here, we present results from a 14-month deployment of a benthic camera platform tethered to the VENUS cabled observatory in the seasonally hypoxic Saanich Inlet. Our time series continuously recorded natural cycles of deoxygenation and reoxygenation that allowed us to test whether a community from the NEP showed hysteresis in its recovery compared to hypoxia-induced decline, and to address the processes driving temporal beta diversity under variable states of hypoxia. Using high-frequency ecological time series, we reveal (i) differences in the response and recovery of the epibenthic community are rate-limited by recovery of the sessile species assemblage; (ii) both environmental and biological processes influence community assembly patterns at multiple timescales; and (iii) interspecific processes can drive temporal beta diversity in seasonal hypoxia. Ultimately, our results illustrate how different timescale-dependent drivers can influence the response and recovery of a marine habitat under increasing stress from environmental change.

## 1. Introduction

Oxygen in the global ocean is predicted to decrease by 2 to 3% over the next century due to the indirect consequences of atmospheric warming [1,2]. Some of the greatest proportional losses will occur in the Northeast Pacific (NEP) where oxygen has historically been low compared to other ocean basins [3,4]. Because of irregular sampling intervals in historical data, the influence of decadal-scale fluctuations and inter-annual variability, regional observations are necessary to understand the primary drivers of the long-term deoxygenation in the NEP [5]. However, our understanding of the ecological consequences due to the changing oxygen regime in the NEP is still constrained by historical observations and thresholds biased towards the Atlantic Ocean [6], where severe hypoxia (O_2_ < 0.5 ml l^−1^) has been absent as an evolutionary pressure [7]. As a result, global hypoxia thresholds (O_2_ < 1.4 ml l^−1^) fail to predict how and when biodiversity will respond to the changing oxygen conditions in the NEP [6,8,9].

The effect of ocean deoxygenation on biodiversity and ecosystem function is a growing global concern [10,11]. Regardless of the environmental drivers of hypoxia, there are ecological consequences once thresholds are exceeded: compression of species distributions as a result of aerobic habitat loss [4,6,12], poleward migration of tropical species [13] and a potential loss in ecosystem biomass [14]. More generally, as oxygen diminishes, species richness (alpha diversity), abundance and biomass all decline [15–18], while species turnover increases due to interspecific differences in hypoxia tolerance [6,18–20]. Thus, beta diversity (the difference in community composition among samples) is high when hypoxia varies across spatio-temporal gradients such as those characterizing the upwelling zones of eastern boundary currents [17]. Most studies have focused on spatial patterns and processes; hypoxic gradients also change in time on various scales: decadal [21,22], seasonal/annual [23,24] and even daily [25]. Although measures of beta diversity in the temporal context are not yet common (but see [26–28]), temporal patterns should reflect rate and frequency of species replacement as habitat suitability shifts. To date, our ability to differentiate short-term versus long-term responses in benthic communities has been limited to annual and seasonal-scale time series [29]. In terms of the rates of marine habitat degradation and recovery, the relative influence of rapid habitat change and community response on shorter timescales is not accounted for in the current trajectories of marine biodiversity loss, which primarily focus on the effects of long-term deoxygenation.

Basin water of Saanich Inlet, a coastal fjord in British Columbia, has an annual hypoxia cycle caused by organic matter breakdown and seasonal oxygen replenishment [30]. Coastal wind patterns cause seasonal upwelling that drives deepwater oxygen renewal and can vary in strength from year to year [31,32]. Oxygen recovers by deepwater renewal in the fall [30] and partial mid-depth renewal in the spring [32]. Thus, weak renewals caused by El Niño-Southern Oscillation or other offshore events can influence the volume of hypoxic water in Saanich Inlet [6,33]. The hypoxia cycle also results in spatial reorganization of the benthic species in Saanich Inlet [6], which occurs at much lower hypoxia thresholds compared to those of other major ocean basins [8]. Since 2006, Ocean Networks Canada's VENUS cabled observatory has monitored seafloor conditions in Saanich Inlet by providing high-frequency environmental data at 10 min or shorter intervals. The advanced subsea technology and natural *in situ* variability in Saanich Inlet make it a model system for examining the temporal processes that can structure marine communities influenced by oxygen deficiency. For example, using camera platforms tethered to VENUS, tidal periodicities (two-week interval) and short-term habitat variability have been shown to characterize the seasonal response of two crustaceans at this site [34,35]. Extensive observations on the behavioural response of one of these crustaceans, the squat lobster *Munida quadrispina*, also suggest that resource availability (i.e. lack of infauna and substratum) can be important in seasonally hypoxic systems [36].

We build on this past work by deploying an updated camera platform to generate a 14-month study acquiring images and concomitant data collected at sub-daily intervals; this set of continuous observations spans temporal periods of both deoxygenation and reoxygenation. Our time series is novel in that it spans phases of oxygen decline and recovery in the NEP; therefore it allows us to test whether hysteresis in recovery is evident in a ‘hypoxia-adapted’ assemblage (i.e. decline and recovery trajectories are different) as documented in systems from the Atlantic Ocean [37,38]. As past studies focused on only a few species, we also examined and compared the response of the entire epibenthic community with individual key species [8] to test if the response to oxygen variability is similar at different biological levels of organization (i.e. population versus community) and whether different environmental factors influence the response at different temporal scales. To facilitate our temporal beta diversity assessment, we applied several novel multivariate statistical methods designed to analyse the multiscale nature of ecological time series [39]. Ultimately, our results illustrate how different timescale-dependent drivers can influence the response and recovery of benthic communities living in highly variable habitat conditions.

## 2. Material and methods

### 2.1. Study site—Saanich Inlet

**Figure 1.**
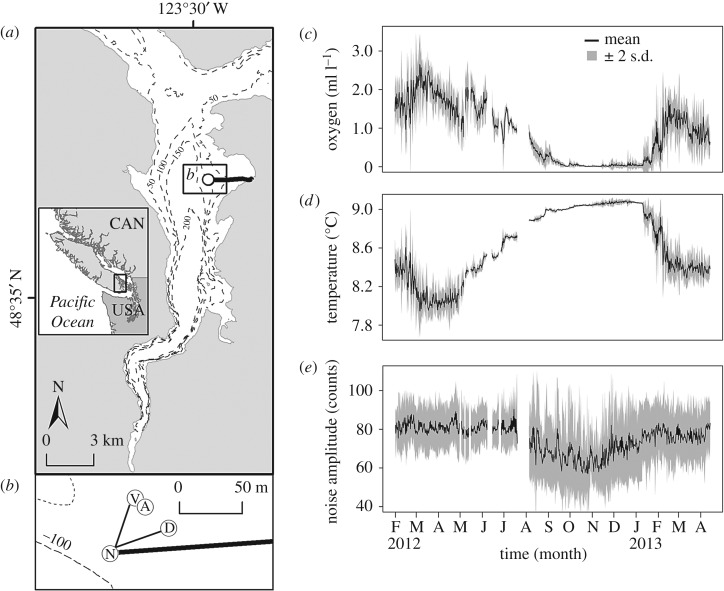
Observatory instrumentation in Saanich Inlet. (*a*) Saanich Inlet is located on Vancouver Island, British Columbia (inset). A surface-to-site power cable (black line) connects to VENUS subsea instrumentation (white circle) at a depth of 96 m. Contours are 50 m intervals. (*b*) The central node (site N) is the interface for power and communication to the main VENUS instrument platform (VIP; site V) and the DISCo camera array (site D), both located at 95 m. The Aquadopp current meter (site A) measuring backscatter is tethered to the VIP. (*c–e*) *In situ* VENUS data capture the magnitude of environmental variability over the annual hypoxia cycle. Data are the mean (black line) ± 2 s.d. (grey band) summarized to match the 12-h interval between imagery timestamps and were calculated from original raw per minute VENUS data. (*c*) Dissolved oxygen and (*d*) temperature were measured at 0.3 m above bottom at site D. (*e*) Backscatter was measured at 1 m above bottom using the return signal strength (noise amplitude) from beam 1 of the current meter at site A.

Saanich Inlet (figure 1*a*) is a highly productive basin [40,41] on Vancouver Island, British Columbia, Canada (figure 1*a* inset) with a maximum depth of approximately 225 m and a shallow entrance sill at 75 m that restricts deep water exchange beyond the inlet. An annual refreshing cycle causes alternating phases of hypoxia expansion and oxygen recovery. Deep anoxic waters in the basin are displaced upwards during the summer, forcing low-oxygen waters into shallower depths and registering as deoxygenation at instruments deployed at mid-depth (approx. 100 m). In the fall and spring, horizontal exchange with outside source waters causes less dense, oxygenated waters to flow over the sill to reoxygenate the basin [6,30]. Intensity and duration of deoxygenation may vary between years [6,35,42]. The epibenthic community in Saanich Inlet is composed of fish and invertebrate species that are common to the adjacent Salish Sea and the continental shelf and slope of the northeast Pacific Ocean. Over the annual hypoxia cycle, community-level changes are driven primarily by the abundance of a few key species [6,35].

### 2.2. Seafloor observatory configuration

The VENUS Observatory of Ocean Networks Canada (ONC, www.oceannetworks.ca) maintains a cabled array in Saanich Inlet. For our study, a seafloor camera platform gathered data in real time with scientist-controlled feedback through the Internet. Automated image capture was possible with ‘DISCo’ (Digital Stills Camera System) that consisted of a digital still camera (modified 8MP CCD Olympus C8080 with an Ikelite 200 W flash) with a pair of horizontal scaling lasers (7 cm) mounted to a Sidus pan and tilt unit (180° tilt range, 360° pan range). A Sea-Bird SBE 16plus model 4996 CTD and an Aanderraa Optode model 4175 oxygen sensor were mounted onto the DISCo frame, and measured water properties 0.3 m above the seafloor (figure 1*b*). Near-bottom acoustic backscatter was sampled at 1 MHz from a Nortek Aquadopp profiling current meter located within 25 m of DISCo (figure 1*b*). We used the backscatter intensity of a single beam as a proxy for near-bottom, suspended particles and zooplankton (e.g. [43]). This narrow beam was oriented parallel to the seafloor at 1 m above the bottom and resolved suspended particles greater than 1 mm in size [44]. The main VENUS instrument platform (VIP) nearby has provided a near-continuous record of water characteristics since 2006. Instruments are powered from shore through a cable that also enables real-time acquisition of images and water data.

DISCo was deployed at a bottom depth of 96 m about 25 m from the VIP in Patricia Bay, Saanich Inlet, where the primary substratum is bedrock covered by a 10–20 cm deep layer of fine silt with cobble. The substratum differs from studies at the 103 m site in Saanich Inlet [34,35] where the poorly consolidated sediments host dense *Beggiatoa* spp. bacterial mats and minimal infauna [43]. The deployment from 20 February 2012 to 06 May 2013 (442 days) encompassed an entire hypoxia cycle (figure 1*c*). On 16 August 2012, DISCo was recovered for maintenance and redeployed by a remotely operated vehicle (ROV) that recreated the position by remotely adjusting pan and tilt settings through ONC online software. The DISCo automated protocol was programmed to collect two images at two non-overlapping areas of the seafloor (two at 30° pan left and two at 30° pan right) every 0.5 h. However, intermittent failures caused 28% of the total sequence (approx. 89 000 images) to be lost, which resulted in data gaps ranging from 1 h to 15 days throughout the deployment. Thus, we designed protocols for imagery processing, imagery analysis and statistical analyses to accommodate this irregular sampling design and preserve a 12-h interval time series with a statistical analysis resolution of 24 h.

### 2.3. Imagery processing and data management

The chronological image order was randomized to reduce observer biases during imagery annotation. Two images (the first from each pan position) were analysed at 00.00 and 12.00 local time. If the first image of a position was missing, the second image was analysed. If both images from a position were missing, both images from the other pan position were analysed. If only one of the four images from a time point was present, it was analysed. If all four images were missing, the images at the closest time point, within 30 min before or after, were analysed following the above steps. Using this protocol, we analysed a total of 1573 images which, due to data gaps, represents 85% of the 12-h interval time series.

In each image, individual epibenthic animals resolvable to 1 cm in size were counted, identified to species when possible or classified to morphotype. Because the field of view was angled towards the seafloor, we used perspective grids that were calibrated with a temporary physical grid laid by the ROV to calculate the area covered in each image [34,35]. Because sediment resuspension events caused by flatfish [43] and high zooplankton densities affected water clarity, a visibility rank was assigned to each image to assess overall image quality, where 1 = ≤25% of the image was clearly visible, 2 = 25–50%, 3 = 50–75% and 4 = ≥75% [35]. Bacterial mat, euphausiid swarms, chaetognath swarms, amphipod swarms, copepod swarms, worm tubes and settling phytoplankton blooms were recorded as presence–absence data. Plankton identity was confirmed from trapped individuals on the nearby VIP platform [45]. Species abundances were standardized to densities (individuals m^−2^) by pooling counts from both images at a time point and dividing by the combined area of the images (2.7 m^2^) covered in the field of view. If only one photo was available at a time point (9% of the time series), densities were calculated from the area covered by a single image (approx. 1.3 m^2^).

Water column and particulates data were collected at a 1-min interval (hereafter, raw data). Because oxygen partial pressure (pO_2_) drives the ecophysiology of hypoxia in water-breathing organisms [46], pO_2_ was calculated by integrating raw CTD data measured at DISCo on salinity, temperature, pressure and density with oxygen concentration values (‘*po2*’ function, [47]); this pO_2_ was used in all statistical analyses. The raw oxygen data had a slight offset (approx. 0.048 ml l^−1^), which manifested as the minimum value in histograms of the measurements. The period of minimum oxygen was interpreted as anoxia as registered by the adjacent VIP oxygen sensor and confirmed through comparisons with multiple sensors and Winkler titrations [42]. Therefore, we subtracted 0.048 ml l^−1^ from the entire oxygen time series (e.g. [42]) before proceeding with further analyses. For comparability, results are presented in the concentration unit ml l^−1^, which is most commonly used in biological studies. As temporal variability can affect how epibenthic organisms respond to environmental conditions [35,48], raw data for oxygen, temperature and particulates were summarized into mean, maximum, minimum and standard deviation [35] for the 12-h interval preceding the image timestamps and used as explanatory variables in the community analyses. Data processing and statistical analyses were done in R v.3.0.2 [49].

### 2.4. Statistical analyses: temporal changes in species diversity

Species richness (Shannon–Weiner diversity index, $H_{\ln}^{\prime }$) and evenness (Pielou's Evenness Index, J) were used to assess alpha diversity at each time point. Beta diversity assessed compositional heterogeneity among time points. We calculated beta diversity following the variance partitioning framework of Legendre & De Cáceres [50] using the R function ‘*beta.div*’*.* This approach is advantageous in temporal studies because each time point has its own ‘local’ value compared to other methods [40] that calculate beta diversity from pairwise site comparisons. For this method, total beta diversity (BD_TOT_) among all time points is equal to the total sums of squares in the species composition matrix. BD_TOT_ can then be partitioned into the relative contributions from each time point (LCBD, local contribution to beta diversity) or by individual species (SCBD, species contribution to beta diversity) [50]. This novel method allows alpha and beta diversity to be directly compared by mapping index values at the same intervals on the same *x*-axis (time). Large LCBD values indicate time points when the composition of species is unique relative to the entire time series because all sampling points are compared to a mean value. Species (*n* = 41) abundance data were Hellinger-transformed and redundancy analysis (RDA) examined species relationships over the time series. By using the sequential timestamp and a second-order polynomial of the timestamp as explanatory variables [51], an ordination plot could then be used to visualize the sequential turnover of the community and assess whether species replacement (i.e. new species invading) or species recovery (i.e. same species returning) was occurring over the hypoxia cycle [52]. Species turnover rates relative to the hypoxia cycle were calculated with piecewise regression. Models were calculated independently for mean oxygen, total abundance, abundance of mobile species and abundance of sessile species. Model selection was determined using the Akaike information criterion (AIC) after sequential forward addition of break points to the model. The slope of each line in the final model was used to calculate relative rates of change in environmental oxygen concentration ([O_2_]_env_), mobile species assemblage abundance, sessile species assemblage abundance and total abundance during the hypoxia cycle. Piecewise regression was done using the R package ‘*segmented*’ [53].

### 2.5. Temporal drivers of community structure

Distance-based Moran's eigenvector maps (dbMEM) were used to assess the environmental variables influencing species composition at the different timescales in our study (see Supplementary methods in the electronic supplementary material for details). Species (*n* = 41) abundance data were first Hellinger-transformed to avoid overweighting rare species [54] and significant linear trends were removed. To accommodate the 15-day gap between deployment periods and still perform a single global analysis for the entire 14-month time series, we generated a staggered dbMEM matrix for our analyses [55]. To reduce the other, smaller data gaps (excluding the 15-day gap) to a maximum interval of 24 h, supplementary time points (*n* = 19) were added to the time series [56] prior to generating the dbMEM variables for the two different deployment periods. The addition of these supplementary time points does not affect the dependent variables [56] but allows fine-scale temporal structure to be represented better in the analysis that would otherwise be constrained to the size of the largest data gap. The trade-off is a slight loss in orthogonality among the eigenfunctions which prevents reducing the sampling interval any further. All supplementary time points were removed prior to using RDA involving species abundance data.

Separate global dbMEM analyses were performed on the multispecies and univariate key-species (slender sole, squat lobster) abundance data to compare differences in their response to the hypoxia cycle. Variance partitioning on the dbMEM variables and environmental variable matrix [51] informed on the relative contribution of positive temporal correlation (positive time dependence), negative temporal (negative time dependence) correlation and environmental drivers in explaining variation in the biological response. By accounting for temporal correlation, influential environmental and biological processes that are not included in the matrix of predictor variables can be captured by the dbMEM analysis [39].

To assess fine-scale temporal patterns, dbMEM submodels were developed with the dbMEM eigenfunctions modelling positive autocorrelation from the second deployment period which had fewer data gaps (see Supplementary methods in the electronic supplementary material). Submodels representing broad, medium and fine scales (relative to the second deployment period) were used to determine if variation in the community and key species response could be explained by different environmental variables at different timescales (see Supplementary methods in the electronic supplementary material). Fitted species values generated from an RDA of the biological response data with the dbMEMs of each model were used in regressions with a matrix of explanatory environmental variables (*n* = 25). Environmental variables included the dummy-coded visibility ranks (*n* = 6), biological drivers as presence/absence data (*n* = 7), mean values and measurements of variability (s.d., max, min) for oxygen (*n* = 4), temperature (*n* = 4) and suspended particulates (*n* = 4). Model simplification was done by AIC stepwise regression which maximized explanatory power (_adj_*R*^2^) and parsimony [39]. Variance inflation factors (VIFs) were checked among predictors using the ‘*car*’ R package; final sets of predictors in all models had VIFs ≤ 10. Retained dbMEM eigenfunctions with negative Moran's I values were not subdivided into submodels because of the relatively low _adj_*R*^2^ value for the global models (_adj_*R*^2^ = 0.02–0.03). Multivariate analyses, variance partitioning and Venn diagrams were done using the ‘*vegan*’ [57] and ‘*packfor*’ R packages [58]. dbMEM temporal eigenfunctions were generated using the ‘*PCNM*’ R package [59].

### 2.6. Oxygen trends from the first 11 years of VENUS (2006–2017)

We examined the near 11-year (March 2006 to January 2017) VENUS VIP oxygen time series to set our 14-month observation period in the context of the broader long-term conditions at our study site. Since 2006, *in situ* dissolved oxygen concentration ([O_2_]_env_) has been measured every minute at the VIP at 96 m depth using a sensor that is approximately 1.5 m above the seafloor. The almost 11-year period of March 2006 to January 2017 was analysed as an update to the original analysis from Chu & Tunnicliffe [6]. Intermittent data gaps were linearly interpolated before calculating 10-year trends. Because of the sinusoidal pattern of the hypoxia cycle, the start and end of the time series were truncated at the 2006 (16 March 2006) and 2016 (27 May 2016) oxygen maximums before a 10-year linear trend was calculated. A 1-year running mean was used to determine the annual trend over the 10-year period [6]; six months were removed from the start and end of the complete one-year running mean because of boundary effects of the calculation. The cumulative, annual duration (from March to March for 2006–2016, and from March to January for 2017) was calculated, at which [O_2_]_env_ was below the 0.5 ml l^−1^ severe hypoxia threshold [23] and below the 0.88 ml l^−1^ ecophysiological threshold determined for the East Pacific Ocean [8]. Linear regressions were used to determine if the annual duration below each hypoxia threshold has increased over time.

## 3. Results

### 3.1. Community response in a highly variable environment

The *in situ* magnitude of environmental variability was resolved using the high-frequency VENUS data. Over the study period, the range of daily and hourly fluctuations in environmental oxygen concentration (hereafter [O_2_]_env_) reached approximately 2.7 ml l^−1^ and approximately 2.1 ml l^−1^, respectively (figure 1*c*). The range of daily and hourly temperature fluctuations reached 0.9°C and 0.7°C, respectively (figure 1*d*). High variability coincided with high mean values for environmental variables; variability and mean both diminished during the onset of deoxygenation in June 2012 before reaching minimum values during anoxia in October 2012. Brief pulses of higher variability reappeared in December 2012 although a marked increase in the mean [O_2_]_env_ did not occur until January 2013. Near-bottom temperature gradually increased from approximately 8.0°C to approximately 9.0°C, in parallel with deoxygenation, before decreasing during reoxygenation. A marked decrease in backscatter measured by the Aquadopp beam also occurred during deoxygenation (figure 1*e*), indicating a decrease of near-bottom, suspended particles and zooplankton during severe hypoxia ([O_2_]_env _< 0.5 ml l^−1^). The duration of severe hypoxia lasted for 174 days, which included 94 days when the average oxygen was <0.05 ml l^−1^.

**Figure 2.**
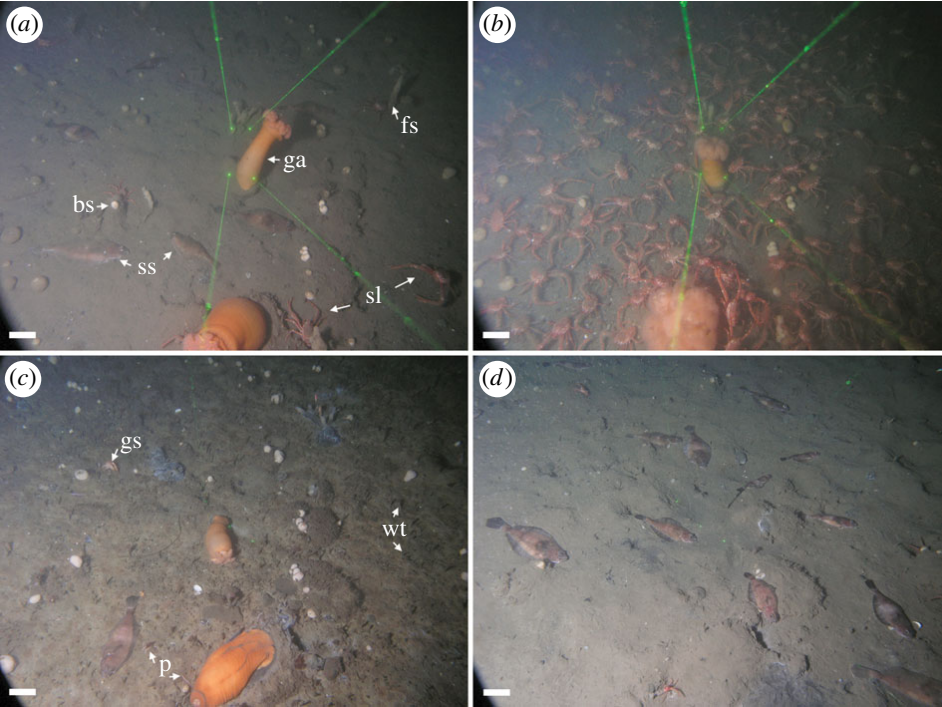
Transitional phases of the epibenthic community in seasonal hypoxia. (*a–d*) Same field of view from DISCo (pan right, area is 1.4 m^2^). (*a*) At the start of the time series (Feb 2012), environmental oxygen concentration ([O_2_]_env_) was 1.9 ± 0.2 ml l^−1^ (mean ± s.d. over 12 h). Slender sole (ss) and squat lobster (sl) were the dominant mobile species. Sessile species included giant anemones (ga), ball sponges (bs), finger sponges (fs) and ascidians. (*b*) A large squat lobster migration event (adult max density = 123 ind. m^−2^) occurred at the onset of deoxygenation in Jun 2012 ([O_2_]_env_ = 1.9 ± 0.3 ml l^−1^). (*c*) By Nov 2012, local contributions to beta diversity increased in near-anoxia ([O_2_]_env_ = 0.05 ± 0.01 ml l^−1^) with the emergence of macroinfauna such as ghost shrimp (gs), mobile epifaunal polychaetes (p) and those that built worm tubes (wt). Slender sole and sessile species were present in reduced numbers. (*d*) After reoxygenation (Feb 2013, [O_2_]_env_ = 0.6 ± 0.3 ml l^−1^), slender sole abundance increased, macroinfauna disappeared, but sessile species abundance decreased due to the hypoxia-induced mortality of the sponges, ascidians and the two giant anemones. Scale bars = 10 cm.

A total of 44 190 sightings of 41 species from eight metazoan phyla were recorded, including presence/absence records of bacterial mats, zooplankton species and worm tubes formed by emergent macroinfauna. The sponges *Aaptos simplex* and *Homaxinella amphispicula* contributed to over 50% of the total species abundance data. Despite the extended period of anoxia, metazoan life was never entirely excluded from the study site as our time-lapse imagery captured the successional turnover of the community during this period (electronic supplementary material, video S1). The epibenthic community was characterized by a mixed assemblage of mobile and sessile species during the period of high [O_2_]_env_ at the start of the time series (figure 2*a*). A large squat lobster migration event occurred in May 2012 when the density of squat lobster reached 123 ind. m^−2^ (figure 2*b*) during relatively high [O_2_]_env_ (mean ± s.d., 1.9 ± 0.3 ml l^−1^). When deoxygenation excluded most of the mobile species, slender sole and squat lobster remained although their overall density decreased in anoxia. During deoxygenation and in anoxia, the community composition shifted to a state characterized by a mixed assemblage of sessile species and emergent macroinfauna when callianassid shrimp *Neotrypaea californiensis* and tubiculous polychaetes emerged from the sediment (figure 2*c*; electronic supplementary material, video S1). Sessile species such as two giant anemones *Metridum farcimen*, the sponges *A. simplex* and *H. amphispicula*, and white ascidian *Ascidia* sp. initially survived the exposure to anoxia, but then underwent mass mortality after [O_2_]_env_ had already recovered to higher levels ([O_2_]_env_ = 0.6 ± 0.3 ml l^−1^), which illustrates the time delay between population decline and the initial onset of anoxia. Habitat reoxygenation coincided with the disappearance of the emergent macroinfauna and an increase in slender sole abundance (figure 2*d*).

### 3.2. Compositional turnover in the epibenthic community

**Figure 3.**
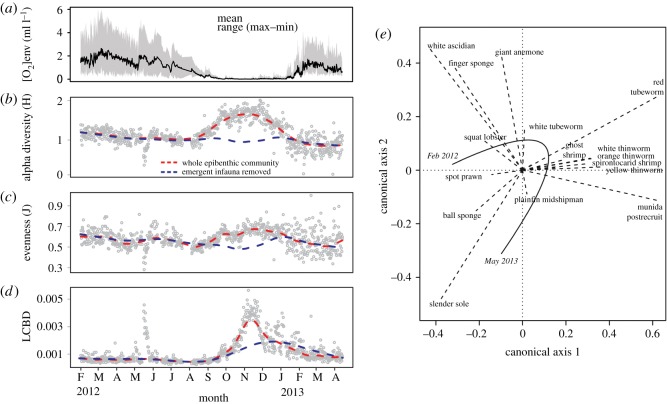
Compositional heterogeneity in seasonal hypoxia. (*a*) Environmental oxygen data ([O_2_]_env_) are summarized from per minute data for the 12-h interval before each sampling time point from February 2012 to March 2013. The annual hypoxia cycle is characterized by relatively higher average [O_2_]_env_ and variability before and after the onset of severe hypoxia ([O_2_]_env_ mean < 0.5 ml l^−1^). Temporal changes in (*b*) alpha diversity (${\text{H}}_{\ln}^{\prime }$), (*c*) species evenness (J) and (*d*) local contributions to beta diversity (LCBD) during seasonal hypoxia. Dashed lines are smoothing curves through the sampling time points (circles) that illustrate general trends of the epibenthic community with the emergent infauna included (red) or excluded (blue). May 2012 peaks in alpha diversity, evenness and LCBD were a result of a mass migration of squat lobster (figure 2*b*). (*e*) Ordination plot of the species (dashed lines) relationships to time (curved line with the start and end dates) shows the sequential importance of species in driving temporal beta diversity. Species clustered near the origin (0,0) were removed to improve clarity. Slender sole are strongly correlated with the beginning and end of the time series. Spot prawn are associated only with the beginning of the time series (vector points towards start) because they do not return before the end of the observation period.

Marked shifts in species diversity and composition corresponded to the shifting [O_2_]_env_ during the hypoxia cycle (figure 3*a*). Alpha diversity (${\text{H}}_{\ln}^{\prime }$) remained constant throughout periods of high [O_2_]_env_, increased during the onset of severe hypoxia in September 2012 and reached maximum diversity during the [O_2_]_env_ minimum in November 2012 as a result of emergent macroinfauna (figure 3*b*). In contrast, species evenness (J) was relatively constant relative to the marked shifts in the diversity indices throughout the seasonally shifting [O_2_]_env_ (figure 3*c*).

By tracking values of local contribution to beta diversity (LCBD) to sampling time points, we identified the periods when marked changes in compositional heterogeneity occurred. Low values of LCBD indicated that the species composition was similar at the beginning and end of the time series (figure 3*d*). High values of LCBD identified two different periods when the species assemblage was distinct relative to the ‘average’ community composition. The first period occurred over three weeks in May 2012 as a result of the mass migration of squat lobster which also corresponded to decreases in alpha diversity and species evenness (figure 3*b*,*c*). The complete coverage of the seafloor by squat lobster probably obscured sessile species in the images during this brief period. The second period occurred over four months from October 2012 to February 2013 when deoxygenation induced the exclusion of mobile megafauna, emergence of infauna and death of most sessile fauna. The resulting increase in the relative proportion of mobile species within the epibenthic community manifested as a general increase in LCBD variability at the end of the time series (figure 3*d*).

Values of species contribution to beta diversity (SCBD) for 11 species were well above the mean of the community assemblage: the spirontocarid shrimp *Spirontocaris sica*, two demosponge species (*H. amphispicula*, *A. simplex*), four emergent macrofauna species, white ascidian, slender sole *Lyopsetta exilis*, and both adult and post-recruit squat lobster *Munida quadrispina*. Slender sole and both life stages of squat lobster had the highest SCBD values among species (an order of magnitude greater than the mean SCBD index value), which highlights their importance in driving compositional heterogeneity within the local community. The contribution of slender sole to temporal beta diversity can also be seen in the RDA ordination plot, where the community composition before and after deoxygenation is primarily driven by the abundance of slender sole (figure 3*e*).

### 3.3. Rates of community decline and recovery in hypoxia

**Figure 4.**
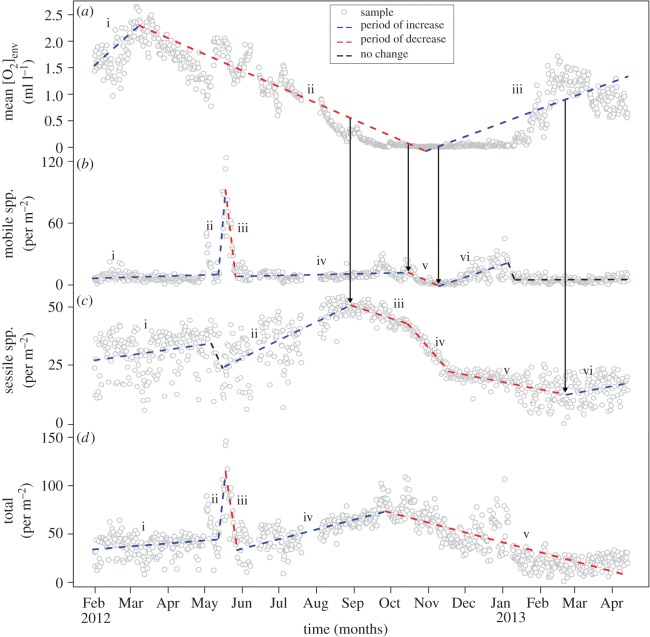
Community response and recovery rates in seasonal hypoxia. Piecewise regression determined temporal transition points and intervals of change in (*a*) mean [O_2_]_env_ and the abundance of (*b*) mobile species, (*c*) sessile species and (*d*) the total community. Dashed lines indicate whether intervals significantly increased (95 CI of slope > 0), decreased (95 CI < 0) or had no change (95 CI overlaps with 0) over time. Roman numerals indicate intervals with a significant rate and are summarized in table 1. Arrows indicate the points in the hypoxia cycle when oxygen-driven changes occurred in the abundance of mobile and sessile species. The interval over which mobile fauna declined (*b*, v) was brief compared to that of sessile fauna (intervals *c*, iii–v). A decrease in sessile species continued even during reoxygenation of the habitat. As a result, the total abundance of epibenthic animals continued to decline until the end of the time series. Note that the rapid change in abundance observed in May (ii–iii) for (*b*) and (*d*) was caused by the mass migration of squat lobster.

Piecewise regression revealed transition intervals and relative rate changes of the species assemblage during seasonal hypoxia. [O_2_]_env_ declined from a maximum in March 2012 until reaching anoxia in October 2012 before the onset of reoxygenation (figure 4*a*). The interval over which species abundance declined as a result of deoxygenation differed between sessile and mobile species. Although deoxygenation occurred over seven months (figure 4*a*, ii), mobile species abundance declined for only one month, centred on the November 2012 [O_2_]_env_ minima (figure 4*b*, v), at a rate of 0.52 ind. m^−2^ day^−1^ (table 1). The relatively quick response of the mobile species community to reoxygenation was driven by the return of slender sole during the onset of short-term [O_2_]_env_ variability (figure 4*b*, vi). In contrast, sessile species decreased at a rate of 0.17 ind. m^−2^ day^−1^ (table 1) over two months before the [O_2_]_env_ minimum (figure 4*c*, iii) and continued to decrease at rates of 0.1 to 0.6 ind m^−2^ day^−1^ for four months (table 1; figure 4*c*, iv–v) after the onset of reoxygenation (figure 4*a*, iii). The timing and rate of decline in the sessile species community were driven by the gradual hypoxia-induced mortality of sponges, ascidians and anemones. As a consequence, total animal abundance continued to decrease during reoxygenation (figure 4*d*, v) and did not recover before the end of the time series. In general, the rates at which mobile, sessile and total species abundances decreased during deoxygenation were greater relative to rates during reoxygenation (table 1).

**Table 1.** Rates of oxygen-induced community changes in seasonal hypoxia. Rates of decline are negative. Roman numerals refer to line segments in figure 4.

time period		mean rate	95% CI lower	95% CI upper
environmental oxygen – (ml l^−1^ day^−1^)				
i	2012-Feb–2012-Mar	0.02	0.01	0.03
ii	2012-Mar–2012-Nov	−0.01	−0.01	−0.01
iii	2012-Nov–2013-May	0.01	0.01	0.01
mobile species abundance (no. m^−2^ day^−1^)				
i	2012-Feb–2012-Jun 1	0.03	0.01	0.07
ii	2012-Jun 1–2012-Jun 6	14.08	11.98	16.13
iii	2012-Jun 6–2012-Jun 15	−10.11	−11.34	−8.88
iv	2012-Jun 15–2012-Nov	0.03	0.01	0.04
v	2012-Nov–2012-Dec	−0.52	−0.73	−0.30
vi	2012-Dec–2013-May	0.39	0.32	0.45
sessile species abundance (no. m^−2^ day^−1^)				
i	2012-Feb–2012-Jun	0.08	0.04	0.11
ii	2012-May–2012-Sep	0.25	0.22	0.28
iii	2012-Sep–2012-Nov	−0.17	−0.26	−0.08
iv	2012-Nov–2012-Dec	−0.60	−0.75	−0.45
v	2012-Dec–2013-Mar	−0.10	−0.13	−0.07
vi	2013-Mar–2013-May	0.10	0.02	0.18
total species abundance (no. m^−2^ day^−1^)				
i	2012-Feb–2012-Jun 11	0.10	0.04	0.16
ii	2012-Jun 11–2012-Jun 24	11.70	7.03	16.36
iii	2012-Jun 24–2012-Oct	−8.26	−10.36	−6.15
iv	2012-Oct–2013-Mar	0.33	0.28	0.38
v	2013-Mar–2013-May	−0.33	−0.36	−0.31

### 3.4. Timescale-dependent drivers of community structure

Community- and species-level variation were temporally structured by different environmental and biological processes operating at different timescales. Together, the results of the dbMEM analyses revealed significant structure in the species assemblage at multiple timescales. The global dbMEM analyses were significant in modelling positive correlation (_adj_*R*^2^ = 0.57, *p* < 0.001) and negative correlation (_adj_*R*^2^ = 0.01, *p* < 0.01) in the temporal response at the community level. For slender sole, positive correlation models (_adj_*R*^2^ = 0.47, *p* < 0.01) and negative correlation (_adj_*R*^2^ = 0.03, *p* < 0.01) models were also significant. Only the positive temporal correlation model was significant for squat lobster (_adj_*R*^2^ = 0.17, *p* < 0.001). Among dbMEM models developed for the second deployment period, most of the variation was generally explained at the broad timescales (_adj_*R*^2^ = 0.13–0.31, *p* < 0.001) resolved in the time series (table 2; electronic supplementary material, tables S1 and S2). However, significant variation was also explained at the medium (_adj_*R*^2^ = 0.01–0.32, *p* < 0.001) and fine timescales (_adj_*R*^2^ = 0.04–0.11, *p* < 0.001) of the dbMEM sub-models.

**Table 2.** Significant multiscale drivers of community structure. Significant dbMEMs, _adj_*R*^2^ and *p*-values are summarized for the models of global positive (+ve) and negative autocorrelation (−ve), and for the sub-models (second deployment period) of positive autocorrelation at broad (B, 172–53 days), medium (M, 26–11 days) and fine (F, 7.5–2.7 days) scales. The entire species abundance matrix was used as the response variable. Final explanatory variables include biological (bacterial mats (BAC), amphipods (AMP), euphausiids (EUP), copepods (COP), worm tubes (WT) and chaetognaths (CHA)) and environmental (oxygen, temperature and backscatter) drivers. Explanatory variables never retained in models were visibility ranking, oxygen maxima and settling phytoplankton blooms.

				*p*-values of significant explanatory variables																
model summary				biological drivers (presence/absence)						oxygen (kPa)			temperature (°C)				backscatter (counts)			
global	dbMEMs	_adj_*R*^2^	*p*-value	BAC	AMP	EUP	COP	WT	CHA	mean	s.d.	min	mean	s.d.	max	min	mean	s.d.	min	max
−ve	61	0.03	**						*											
+ve	87	0.58	***	***	*	**		***			***			***			***			*
sub-models																				
B	10	0.31	***	***				***			***			***			***	***		
M	28	0.30	***	***				*		***			***				***	***		*
F	49	0.11	***	*			*							***			***		**	

***p < 0.001.

**p < 0.01.

*p < 0.05.

At the community level (*n* = 41 species), the global positive temporal correlation model developed from the second deployment period was significant in explaining half of the variation in species composition (87 dbMEMs, _adj_*R*^2^ = 0.58, *p* < 0.001). Eight of the explanatory variables were significant in explaining community variation: presence/absence of bacterial mats and several zooplankton groups, averages for backscatter and several measurements of variability for oxygen, temperature and backscatter (table 2). The global negative temporal correlation model was also significant (61 dbMEMs, _adj_*R*^2^ = 0.03, *p* < 0.01) and linked a small degree of oscillation in the biological community with suspended particulates and zooplankton (table 2). Although significant explanatory variables were slightly different among the three sub-models compared to the global model, variables always included the presence/absence of certain zooplankton groups and measurements of variability (max, min and/or s.d.) for oxygen, temperature and/or backscatter (table 2).

Individual dbMEM models developed for slender sole and squat lobster revealed differences in the responses of key species to environmental variability. Similar to the community-level analyses, variations in slender sole and squat lobster abundance were structured by different environmental variables operating at multiple timescales (electronic supplementary material, tables S1 and S2). Explanatory variables of slender sole and squat lobster abundance at the global and sub-model levels always included presence/absence of certain zooplankton groups and some measurement of variability for oxygen, temperature and/or backscatter. For slender sole, both the global positive temporal correlation (*n* = 67 dbMEMs, _adj_*R*^2^ = 0.44, *p* < 0.01) and negative temporal correlation (*n* = 119 dbMEMS, _adj_*R*^2^ = 0.03, *p* < 0.01) models were significant (electronic supplementary material, table S1). For squat lobster, explanatory power of the global model was lower than those of the community and slender sole (electronic supplementary material, table S3, *n* = 62 dbMEMS, _adj_*R*^2^ = 0.12, *p* < 0.0001). The negative temporal correlation model was not significant in explaining variation in squat lobster abundance.

### 3.5. Variance partitioning the response of key species

**Figure 5.**
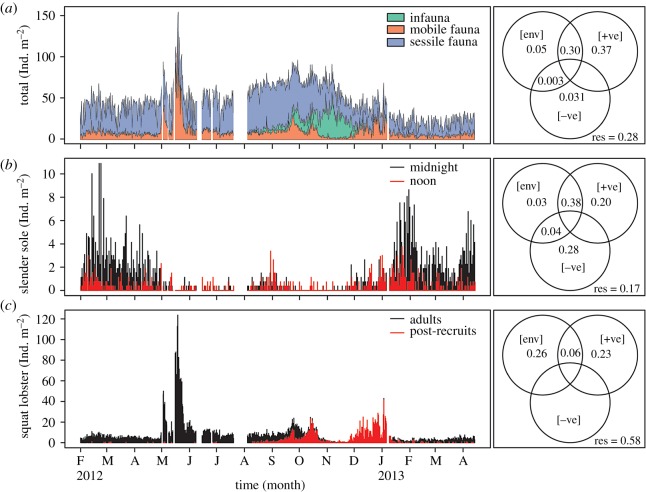
Variance partitioning of the relative influence of environment and temporal autocorrelation on community structure. Venn diagrams represent total variance explained at the (*a*) community or (*b*,*c*) individual key-species level. Overlapping circles represent variance explained by environmental variables ([env]), positive temporal correlation ([+ve]) and negative temporal correlation ([−ve]). Adjusted *R*^2^ (_adj_*R*^2^) values were used to compute fractions of variance. Negative _adj_*R*^2^ values are not shown. (*a*) A stacked area plot illustrates that a substantial portion of community structure is explained by [+ve] and the [env] (_adj_*R*^2 ^= 0.72). The community analysis was performed on the entire species assemblage (*n* = 41 species). For visual clarity, these data were pooled into the three lifestyle modes before plotting to emphasize the general timing of community turnover. (*b*) Overlapping high-density plots of daily slender sole abundances at noon and midnight illustrate a diurnal pattern that is associated with a large proportion of variance explained by [−ve] (_adj_*R*^2^ = 0.28). (*c*) The high abundance of squat lobster occurs during deoxygenation and is associated with a larger proportion of variance explained by [env] that does not overlap with [+ve] (_adj_*R*^2^ = 0.26).

Variance partitioning determined the relative contributions of the environment and temporal autocorrelation in explaining community structure, and highlights the biological processes that influence species assembly patterns. Of the total community variation, 75% was explained by the combination of environment predictors and the dbMEM eigenfunctions modelling positive and negative temporal correlation (figure 5*a*); 72% of the community variation was explained by the environment and positive temporal correlation, revealing a general synchronization of the community response to the seasonal hypoxia cycle; 40% of the community variation was explained by positive and negative autocorrelation (figure 5*a*) that can be attributed to environmental processes not accounted for in the predictors and the influence of biological processes (e.g. species interactions) on community structure.

The influence of biological processes on species assembly patterns was analysed further by applying variance partitioning to the dbMEM models developed for both key species. Of the total, 28% of the variation in slender sole abundance was explained by negative temporal correlation (figure 5*b*). Negative autocorrelation can indicate differences among closely sampled points. The high degree of negative temporal correlation associated with slender sole abundance can be explained by the fine-scale detection limit imposed by the sampling design (24-h) and the type II diurnal (12-h) response [60], where more flatfish were observed at midnight compared to noon during the oxygenated phases of the hypoxia cycle (figure 5*b*). This type II migration pattern was also observed in replicate ROV surveys over a 24-h period in Saanich Inlet when more slender sole occurred deeper (120–100 m) in the evening compared to daylight hours (electronic supplementary material, table S3).

For squat lobster abundance, a greater proportion of variation (26%) was explained by just the environment (figure 5*c*). Minimal overlap between the environment and positive temporal correlation (6%) indicated an asynchronous response of squat lobster abundance to seasonal reoxygenation. In contrast to the coupled response of the broader community and slender sole with deoxygenation, squat lobster abundance peaked during the low-oxygen period as a result of the May 2012 migration and subsequent recruitment events (figure 5*c*). The mass migration behaviour is partially explained by endogenous processes relating to seasonal reproduction in squat lobster. In a separate but parallel study, captive squat lobsters would undergo mass moulting as a precursor to reproduction in the spring (electronic supplementary material, figure S4). Although these captive squat lobsters did not experience field conditions, moulting was synchronized to the timing of the mass migration events observed in the field ([34], this study).

### 3.6. 11-Year trends in the Saanich Inlet hypoxia cycle

**Figure 6.**
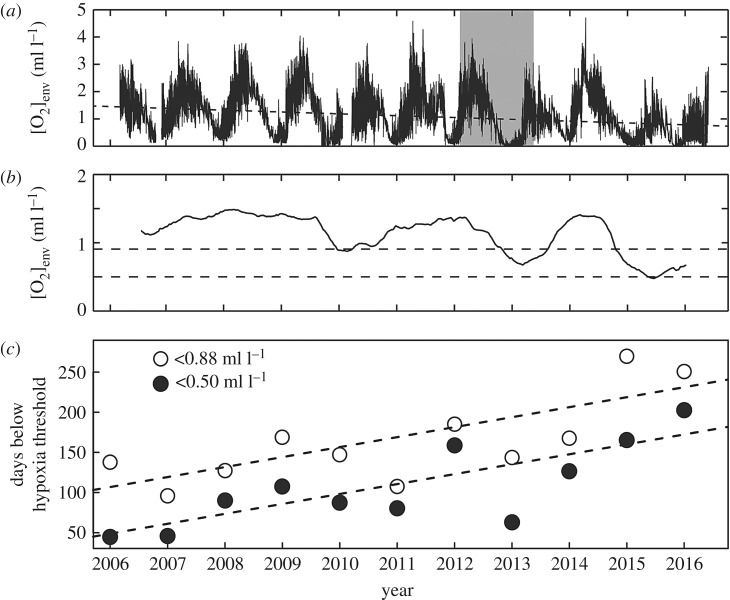
11-year oxygen trends in the Saanich Inlet hypoxia cycle. [O_2_]_env_ from VENUS was measured at minute intervals from March 2006 to January 2017. (*a*) A linear regression (dashed line) shows a significant decadal trend of decreasing [O_2_]_env_ ([O_2_]_env_ = 1.5 − 0.067 × year). Grey band highlights the 14-month deployment period of DISCo. (*b*) One-year running mean shows the decadal oxygen decline is a result of years where there was weakened renewal or enhanced deoxygenation (2010, 2013) and a sustained 2-year period of oxygen depletion (2015–2016). Dashed lines indicate the 0.5 ml l^−1^ severe hypoxia threshold and 0.88 ml l^−1^ ecophysiological threshold for the East Pacific Ocean. (*c*) The annual duration (cumulative number of days starting from March) at which the study site was below the severe hypoxia and critical oxygen thresholds. Dashed lines illustrate the significant trend of increasing duration over time for both the severe hypoxia and critical oxygen thresholds (both, 12.4 days year^−1^). Note the cumulative duration for 2016 was calculated from only nine months at the time of the analysis.

The near 11-year (March 2006 to January 2017) oxygen time series at the study site shows the average [O_2_]_env_ has declined at a significant rate of 0.067 ml l^−1^ year^−1^ (figure 6*a*, *p* < 0.0001), which is higher than the previous rate of 0.05 ml l^−1^ year^−1^ calculated for the first 8 years of VENUS records [6]. This decade-long, negative trend was a result of recent years when average [O_2_]_env_ experienced a marked decline (2010, 2013) due to either a weakened oxygen renewal (e.g. 2010 [6]) or enhanced deoxygenation with a sustained, 2-year period of oxygen deficiency from 2015–2016 (figure 6*b*). As a result, the annual duration at which the system was below biologically relevant hypoxia thresholds has increased by 37% over the past decade. The annual duration of severe hypoxia has significantly increased by 12.4 days per year (figure 6*c*, *F*_1,9_ = 15.57, *p* = 0.003, _adj_*R*^2^ = 0.59) and the duration below the ecophysiological threshold determined for the East Pacific Ocean has also significantly increased by 12.4 days per year (figure 6*c*, *F*_1,9_ = 12.05, *p* = 0.007, _adj_*R*^2^ = 0.53). The trend of annually increasing duration below biologically relevant hypoxia thresholds in Saanich Inlet was not significant during the first 8 years of VENUS [6].

## 4. Discussion

Our study reveals three major features of megafaunal community responses to temporal oxygen gradients: (i) responses to seasonal hypoxia differ at the community and species levels, with the sessile species limiting the rate of community recovery after deoxygenation; (ii) accounting for small temporal scale variability is important when explaining compositional heterogeneity; and (iii) epibenthic communities living in highly variable habitat conditions are structured at multiple time-scales, and both environmental and biological processes influence community assembly patterns. Although the science of using cabled-observatory camera systems has advanced with every successive deployment, technological limitations continue to affect data usability. A substantial amount of data was lost (approx. 28%) owing to technical issues which compromised the resolution of our time series. The resulting irregular sampling intervals and large data gaps can limit ecological interpretation ([35,61,62], this study). Because substantial autocorrelation exists with high-resolution data, analyses must also account for their structured residuals or risk inflating type I error rates and reporting inaccurate explanatory power with classical inferential statistics [63,64]. Despite these constraints, our applied techniques can account for irregular sampling designs and use autocorrelation to explain the ecological processes that structure marine communities.

### 4.1. Differences in the pattern and rates of response in hypoxic communities

The general timing and sequence of community turnover are synchronized with the seasonal period of low oxygen. Most of the variation in the community is explained at the broadest scales reflecting the strong influence of the seasonal hypoxia signal. The natural oscillation between two community phases is driven by the alternating periods of deoxygenation and reoxygenation (also seen in [35]). Deoxygenation excludes most mobile species due to their ecophysiological constraints of aerobic metabolism [8] and induces the death of sessile species under extended exposure. In addition to oxygen variability, factors such as temperature and food sources such as settling phytoplankton blooms and zooplankton influence species composition. The emergence of macroinfauna is probably an escape response to the accumulation of hydrogen sulphide in the sediments that can reach lethal levels during anoxic periods [65]. Shallowing of the inhabitable sediment layer depth is typical of systems with variable states of oxygen deficiency [10,66]. Organic matter supply in addition to oxygen limitation explains macrofaunal community variation in oxygen minimum zones [17,19,67]. However, no single variable can readily predict community turnover patterns in oxygen-deficient systems [19]; this complexity is evident in the different scale-dependent processes and the degree of temporal autocorrelation (i.e. variation not explained by the environmental drivers) revealed by our study design.

The death of the sessile community indicates that benthic systems will experience functional losses in carbon sequestration and nutrient cycling with the loss of suspension and filter feeders [68]. The emergence of macroinfauna would indicate that a further decline in ecosystem function may result from a decrease in bioturbation [69,70]. For example, we consistently observed the callianassid shrimp *Neotrypaea californiensis* above the sediment throughout the low-oxygen phase, which indicates a reduction in the amount of subsurface sediment transported by their burrowing activity [71]. Although high temporal variability can sometimes have a positive effect by mediating functional losses from detrimental shifts in mean environmental values (e.g. temperature) [72], this will not occur in systems influenced by strong deoxygenation because oxygen variability also decreases with the mean.

### 4.2. Interspecific differences influence temporal community dynamics

Interspecific differences in hypoxia tolerance, behaviour and interactions also structure communities in hypoxic systems. While hypoxia-sensitive species migrate to shallower, more oxygenated waters, squat lobster and slender sole show high fidelity to the deeper, severely hypoxic waters (less than 100 m) [6], and can exploit low-oxygen habitats because of their low critical oxygen thresholds (O_2_^crit^) [8]. For these two key community members, oxygen is not the primary limiting factor that drives the *in situ* spatio-temporal dynamics of their populations. For example, repeat observations of juvenile squat lobster recruitment occurring during the anoxic periods in 2008, 2009 and 2012 ([22,70], this study) show a strong coupling of squat lobster reproduction to low-oxygen periods. However, despite extreme hypoxia tolerance and anaerobic capacity [6,8], squat lobster do need to migrate into shallower depths to access the higher oxygen levels needed for mating and moulting in crustaceans [73].

Although slender sole are common in demersal fish assemblages in the northeast Pacific [74,75] and the most abundant ichthyoplankton species in the Salish Sea [76], our study is the first to reveal their diurnal behaviour. As planktivores, slender sole feed on the diel vertically migrating euphausiid *Euphausia pacifica* [77] that are present in Saanich Inlet throughout the year [25]. However, *E. pacifica* are limited by the hypoxia boundary depth [78] that experiences seasonal shoaling in Saanich Inlet [6]. Thus, slender sole probably migrate upslope during daylight hours to feed on descended zooplankton and return to the hypoxic deepwaters at night for refuge. Similarly, food availability in the eastern boundary region of the Benguela current drives analogous diurnal behaviour in the hypoxia-tolerant bearded goby *Sufflogobius bibarbatus* [79,80]. Sediment resuspension and transport as a result of slender sole movement contribute substantially to benthic biogeochemical cycling in this system [43]. If the availability of prey influences migration patterns, then an increased duration of the seasonal low-oxygen period will indirectly affect benthic nutrient cycling through a reduction in the net contribution from slender sole [81].

A lagged recovery in total community abundance (i.e. hysteresis), in which the recovery rates during reoxygenation were slower than the rates of decline during deoxygenation, was primarily influenced by hypoxia-induced mortality of the sessile community. Basal metazoans such as cnidarians and sponges were the most common and sometimes the most abundant sessile species in Saanich Inlet ([19], this study). In general, hypoxia tolerance is notable in both groups; anemones can tolerate anoxia by exploiting anaerobic metabolism [82,83], whereas sponges have some of the lowest *in situ* oxygen occurrences documented [6]. Despite the general hypoxia tolerance of cnidarians and sponges, their critical exposure times can naturally be exceeded *in situ*. Slow growth rates and large adult sizes are often associated with basal metazoans. Thus, the loss of the large adults due to hypoxia [84] would further influence community hysteresis because it takes longer for this size class to re-establish. In systems where seasonal hypoxia can prune the sessile fauna, as observed in our study, larval recruitment and post-settlement growth will ultimately determine the recovery potential of the epibenthic community which, in turn, will be limited by the reoxygenation phase between periods of low oxygen.

### 4.3. Temporal patterns of beta diversity in seasonal hypoxia

In general, the fine-scale community structure, the significance of short-term variability as predictors of biology, and the overall improvement in explanatory power highlight the importance of acquiring fine-scale observations in highly variable habitats. Our revelation of diurnal behaviour in slender sole highlights an unusual flatfish behaviour that probably went undetected in past studies because of multi-day sampling intervals [35], or observations made only during the low-oxygen phase of the hypoxia cycle [61]. The inclusion of short-term environmental fluctuations can also improve our understanding of the timing of biological events. For example, populations of slender sole will generally occupy waters with oxygen levels close to, but not below, their O_2_^crit^ of approximately 0.36 ml l^−1^ [8]. During our study, the average [O_2_]_env_ did not increase above the O_2_^crit^ for slender sole until the beginning of February 2013. The abundance of slender sole, however, increased at the beginning of January 2013, coinciding with an increase in short-term [O_2_]_env_ variability ([O_2_]_env_ range ∼ 0.4 ml l^−1^). The magnitude, duration and frequency of intermittent exposure to elevated [O_2_]_env_ (i.e. ‘intermittency’ [85]) probably allowed slender sole to reoccupy the system although the ‘average’ conditions were still extremely hypoxic (mean [O_2_]_env_ < 0.1 ml l^−1^). Temporal patterns of beta diversity mirror those established in spatial gradients of low oxygen [6] in that compositional heterogeneity is associated with species identity. The occurrence of similar species assemblages before and after the extended anoxic period, the recurrence of slender sole at the end of the time series, and the abundance of squat lobster primarily during low oxygen all indicate that biological context is needed when linking community response patterns to variable states of oxygen deficiency.

Episodic or seasonal hypoxia now appears in estuaries, continental shelves and nearshore margins at sites previously characterized by higher and more stable oxygen regimes [86–88]. In systems where the state of hypoxia has become chronic or sustained, the concept of ‘regime shift’ is ascribed to the community-wide collapse induced by oxygen deficiency (e.g. the Black Sea [89,90]). Because a few tolerant species dominate communities in low-oxygen regimes, the hypoxia thresholds of these key species will determine the critical tipping point [91] at which continued deoxygenation will exclude all higher-level megafauna. While the decadal driver of deoxygenation in Saanich Inlet remains to be confirmed, the trend mirrors those established throughout the upwelling-influenced region of the northeast Pacific Ocean [6]. Response and recovery of the resident marine communities in this region will be affected by the severity, frequency and duration of hypoxic exposure [10,37,92]. For seasonally hypoxic systems, a regime shift will occur if the frequency and duration below critical oxygen levels exceed the time needed for the system to recover through recruitment. As the trend recorded by VENUS sensors continues, a potential hypoxia-induced regime shift in ecosystem structure and function may occur if the duration of hypoxic exposure (i.e. period below threshold points) exceeds the recovery period.

## Supplementary Material

Supplementary Materials

## Data accessibility

Data are deposited at Dryad: https://doi.org/10.5061/dryad.4p9v048 [93]. Supplementary materials have been uploaded as part of the electronic supplementary material.
