# Supplementary material for: Drivers of temporal beta diversity of a benthic community in a seasonally hypoxic fjord
Source: R Soc Open Sci. 2018 Apr 18;5(4):172284. doi: 10.1098/rsos.172284 (PMC5936942; doi:10.1098/rsos.172284)
Supplement: Supplementary Materials [file rsos172284supp1.docx]

**SUPPLEMENTARY MATERIALS**

**Supplementary Methods**

*Distance-based Moran’s eigenvector maps (dbMEM)*

dbMEM analyses were originally developed to model autocorrelation and determine the scale-dependent drivers of community variation under a spatial context [1–3]. These analyses have since been modified and applied to benthic time-series [4–6]. A dbMEM analysis first creates a truncated matrix of Euclidean distances from the observation points in a time series, which are analogous to sampling locations along a spatial transect in one dimension [2]. A principal coordinate analysis (PCoA) is applied to the truncated distance matrix; the resulting principle coordinates are orthogonal temporal eigenfunctions and describe all the time scales resolved in the sampling design. These dbMEM variables can then be used as predictors to determine the time scales at which the species assemblage is structured and the environmental variables that explain variation at those scales [3].

*Accommodating irregular data gaps in time-series*

To accommodate the maximum 15-day gap in the time-series and to use one global dbMEM analysis to examine temporal structure for the entire 14-month hypoxia cycle, a staggered dbMEM matrix was created with separate blocks of MEM eigenvectors [2,7] for each deployment period on either side of the 15-day gap. To reduce the other, smaller data gaps (excluding the 15-day gap) to a maximum interval of 24 hours, supplementary time points (n=19) were added to the time series [8] prior to generating the dbMEM variables for the two different deployment periods. Most of the supplementary time points (n=16) were inserted to fill data gaps during the first deployment period. The addition of these pseudo time points prior to the PCoA does not affect the dependent variables [8] but allows fine-scale temporal structure to be better represented in the analysis that would otherwise be constrained to the size of the largest data gap (15 days in our case). The tradeoff is a slight loss in orthogonality among the eigenfunctions. All supplementary time points were removed prior to using analyses involving species abundance data.

Standard practice with dbMEM analysis focuses only on dbMEM variables with positive Moran’s I. However, dbMEM variables with negative Moran’s I, or negative autocorrelation, were modeled separately from those with positive Moran’s I because our preliminary assessment suggested that negative temporal correlation occurred in abundances of one of the key species in our community (the slender sole, *Lyopsetta exilis*) where values of proximal observations were less similar than those far apart [3].

*Fine-scale sub models*

Because the second deployment period had fewer data gap issues, dbMEM sub-models were developed for this period to examine temporal structure at finer time scales. dbMEM scalograms [8] were made to create sub models by visually grouping positive Moran’s I values generated for the community (n=87 dbMEM variables), slender sole (n=119), and squat lobster (n=62) analyses (Supp. Fig. S1-S3). Although the grouping of the dbMEM variables can be slightly arbitrary [2], the range of broad to fine time scales is captured in ascending rank order of the dbMEM variables. Thus, the sub-model groupings from these analyses are relative representations of the, broad, medium, and fine scales captured by the sampling design [2].. Although the majority of the data were sampled at a 12-hour interval, the fine-scale resolution limit of 24-hour was a result of the data gap issue. Retained dbMEM eigenfunctions with negative Moran’s I values were not subdivided into sub-models because of the relatively low _adj_R^2^ value for the global models. Fitted species values generated from RDA of the species response data and the dbMEMs of each model can then be used in regressions with environmental matrix to determine which predictor variables explain variation at the scale of the associated model.

*Inclusions of variability in sub models*

For the community-level analyses, regressions against environmental data were run twice. First, measurements of variability (s.d., max, min) were excluded for oxygen, temperature, and suspended particulates following methods from Ref. [4]. Second, the complete set of 25 explanatory variables was used. By including measurements of variability, the explanatory power (_adj_R^2^) of the global positive model and sub-models improved by 5% and 2-6% respectively. Therefore, variability measurements were included in all explanatory matrices and only the results of those analyses are presented.

**Figures S1-S3.** Scalograms for subdividing dbMEM variables representing the multiscale temporal structure within the epibenthic community. dbMEM variables (n=501) were generated from a staggered matrix compiled from a time series of observations during the second deployment period of DISCo (August 2012-May 201). N=186 of the dbMEM variables modelled positive temporal correlation (had positive Moran’s I). Supplementary time points (n=3) were added to reduce data gaps to a maximum of 24 hours. dbMEM variables in red were retained from forward selection and visually grouped into sub models representing the relative temporal scales: broad scale (BS), medium scale (MS), and fine scale (FS). Separate scalograms were developed for whole community, slender sole, and squat lobster.


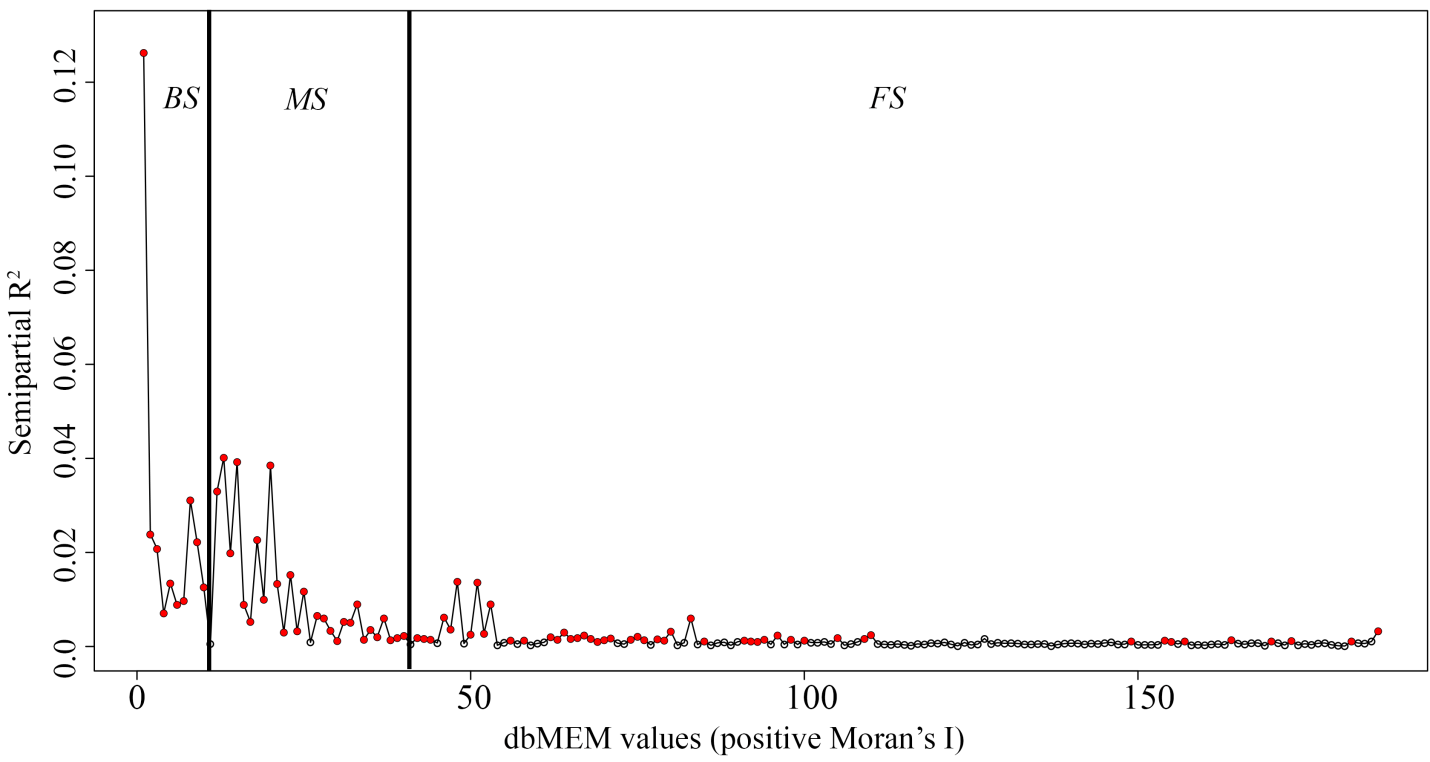


**Figure S1.** Scalograms developed for dbMEM analysis using community matrix (n=42 species) as response data.


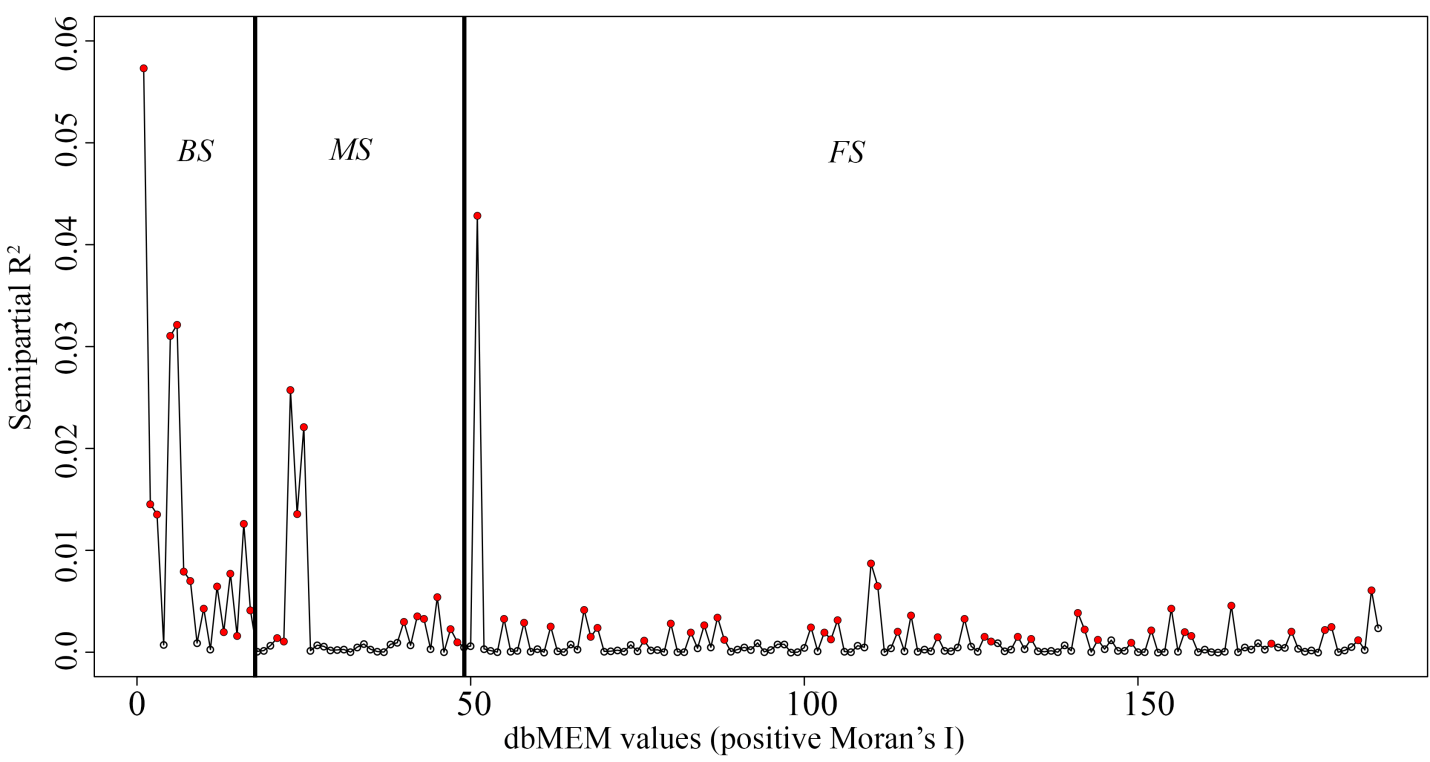


**Figure S2.** Scalograms developed for dbMEM analysis using slender sole abundance as response data.


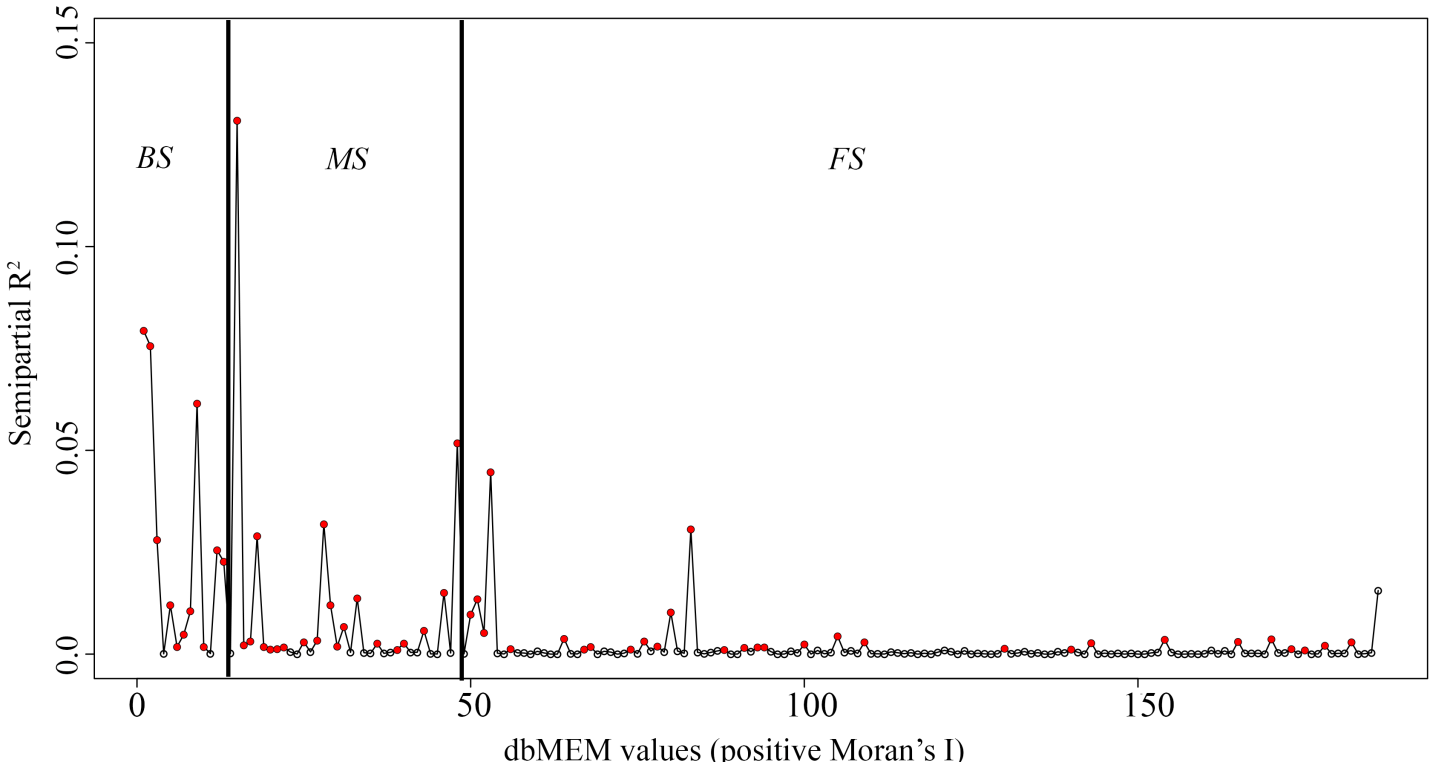


**Figure S3.** Scalograms developed for dbMEM analysis using squat lobster abundance as response data.

**Table S1**. Significant multiscale drivers of slender sole abundance. Significant dbMEMs, _adj_R_2_ and p-values are summarized for the models of global positive (+ve), global negative autocorrelation (-ve) and the nested sub-models of positive autocorrelation at broad (B, 172-12.5 days), medium (M, 10-9.5 days), and fine (F, 9-3 days) scales. Final explanatory variables include visibility ranking (VIS), biological (bacterial mats (BAC), amphipods (AMP), euphausiids (EUP), worm tubes (WT), chaetognaths (CHA)) and environmental (oxygen, temperature, and backscatter) drivers. Explanatory variables never retained in a model were copepods, settling diatom blooms, and temperature standard deviation. *** p <0.001, ** p <0.01, * p<0.05.

|  | | | | P-values of significant explanatory variables | | | | | | | | | | | | | | | | |
| --- | --- | --- | --- | --- | --- | --- | --- | --- | --- | --- | --- | --- | --- | --- | --- | --- | --- | --- | --- | --- |
| Model summary | | | |  | Biological drivers (presence/absence) | | | | | Oxygen (kPa) | | | | Temperature (ºC) | | | Backscatter (counts) | | | |
| Global | MEMs | R^2^ | p-value | VIS | BAC | AMP | EUP | WT | CHA | mean | sd | max | min | mean | max | min | mean | sd | min | max |
| -ve | 67 | 0.02 | *** |  |  | * |  |  | *** |  |  |  |  |  |  |  |  |  |  |  |
| +ve | 119 | 0.44 | *** |  |  | * | * | ** | *** | *** |  |  |  | *** | *** |  | ** | *** | *** | *** |
| Sub-  models | | | |  | | | | | | | | | | | | | | | | |
| B | 14 | 0.31 | *** |  | *** | *** |  | ** |  |  |  | ** | *** |  | * |  | *** |  |  |  |
| M | 11 | 0.32 | *** | ** | *** |  |  | *** | * | *** | * |  |  |  |  | *** | *** | *** |  |  |
| F | 42 | 0.11 | *** |  |  |  | * |  | * |  |  |  |  | *** |  |  | *** | *** | *** | ** |

**Table S2.** Significant multiscale drivers of squat lobster abundance. Summaries of results are presented for the models of global positive (+ve) and the nested sub-models of positive autocorrelation at broad (B, 172-26 days), medium (M, 12-9.5 days), and fine (F, 7.5-3 days) scales. The global –ve autocorrelation model was not significant for squat lobster abundance. Final explanatory variables include biological (copepods (COP) and settling diatom blooms (DIA)) and environmental (oxygen, temperature, and backscatter) drivers. Explanatory variables never retained in a model were bacterial mats, amphipods, euphausiids, worm tubes, chaetognaths, oxygen maximum, oxygen minimum, backscatter maximum, and backscatter minimum.*** p <0.001, ** p <0.01, * p<0.05.

|  | | | | P-values of significant explanatory variables | | | | | | | | | |
| --- | --- | --- | --- | --- | --- | --- | --- | --- | --- | --- | --- | --- | --- |
| Model summary | | | | Biological drivers (presence/absence) | | Oxygen (kPa) | | Temperature (ºC) | | | | Backscatter (counts) | |
| Global | MEMs | R^2^ | p-value | COP | DIA | mean | sd | mean | sd | max | min | mean | sd |
| +ve | 62 | 0.12 | *** | * |  |  |  | *** | *** | *** |  | ** | ** |
| Sub-  models | | | |  | | | | | | | | | |
| B | 10 | 0.14 | *** | ** |  | *** | *** |  | *** | *** |  | *** |  |
| M | 22 | 0.01 | *** |  |  | * |  |  |  |  |  |  |  |
| F | 30 | 0.04 | *** |  | * |  |  |  | *** |  |  |  |  |

**Table S3.** Slender sole *Lyopsetta exilis* were counted from ROV surveys flown along the same section of a benthic transect line at 100 to 120 m depth in Saanich Inlet (for detailed description of methods see Supplementary ref [9]) during night and day (local time). The total abundance is the sum of unburied, partially buried, and buried counts of slender sole. Unburied individuals were 100% above the sediment. Partially buried individuals had 50% of body surface above sediments. Buried individuals had < 10% body surface above sediments.

|  | Night | Day (dawn) |
| --- | --- | --- |
| ROV transect | R1645 – Sep.7, 2013 | R1647 – Sep. 8, 2013 |
| Time (local time zone) | 21:35 to 22:07 | 4:01 to 4:36 |
| **Total abundance** | **518** | **807** |
| Unburied | 297 | 568 |
| Partially buried | 50 | 56 |
| Buried | 171 | 183 |


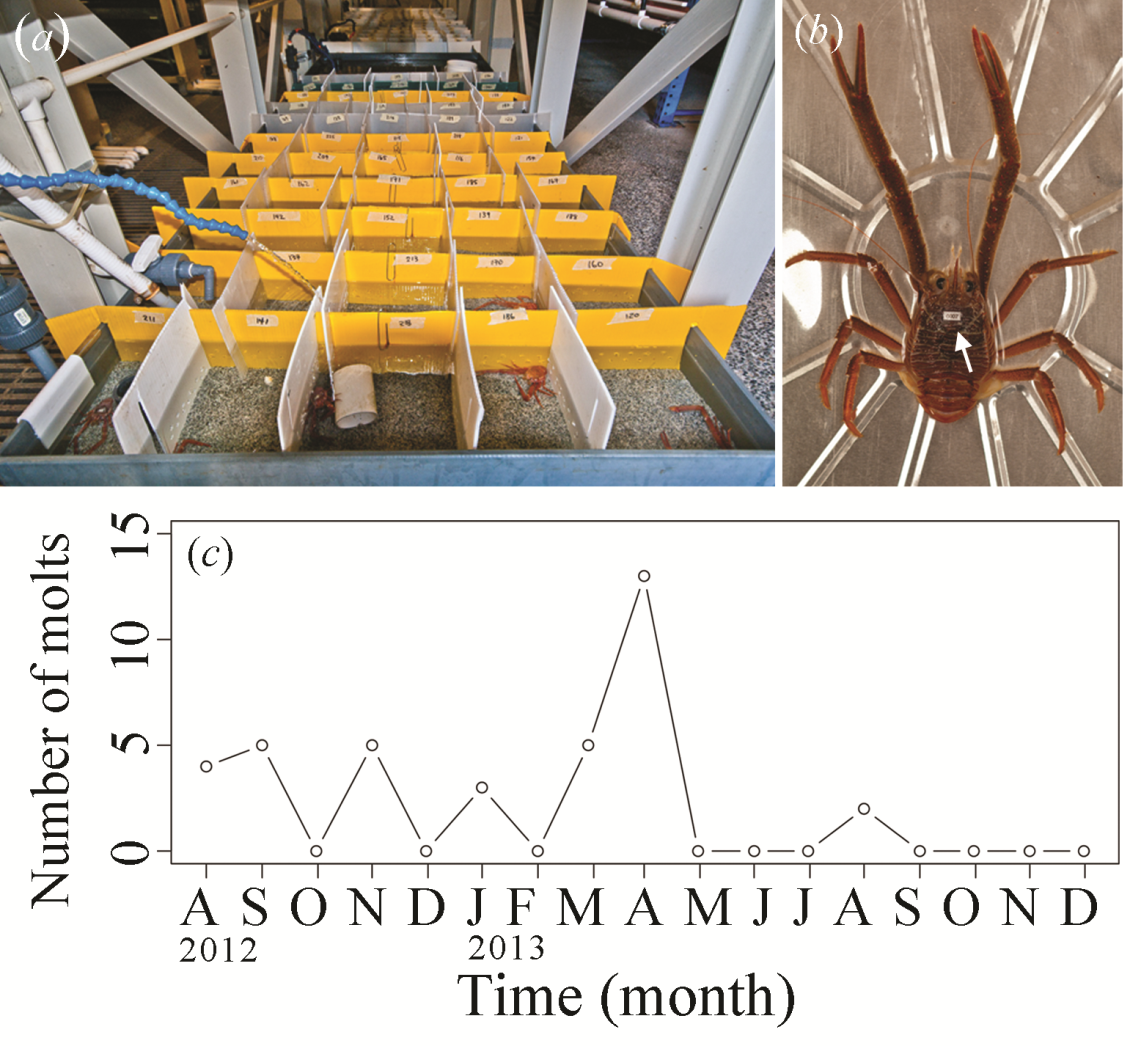


**Figure S4.** A population of squat lobster *Munida quadrispina* was maintained for lab experiments (for detailed description of methods see Ref. [10]). (a) Individually segregated squat lobsters (n=100) were kept in oxygen saturated, recirculating sea water tables and maintained in a perpetually dark, closed system at the University of Victoria from August 2012 – December 2013 and isolated from each other because of cannibalism. Sea water tables were kept under black plastic for the duration of captivity (removed in this image). (b) Individual squat lobsters were tagged with unique identifiers (arrow) which were superglued onto their carapace and retagged after molting. (c) A marked increase in the number of molted squat lobsters occurred in April 2013.

**Supplementary References**

1. Borcard, D. & Legendre, P. 2002 All-scale spatial analysis of ecological data by means of principal coordinates of neighbour matrices. *Ecol. Modell.* **153**, 51–68. (doi:10.1016/S0304-3800(01)00501-4)

2. Legendre, P. & Legendre, L. 2001 *Numerical Ecology 3rd edition*. Elsevier.

3. Legendre, P. & Gauthier, O. 2014 Statistical methods for temporal and space-time analysis of community composition data. *Proc. R. Soc. B Biol. Sci.* **281**, 20132728–20132728. (doi:10.1098/rspb.2013.2728)

4. Matabos, M., Tunnicliffe, V., Juniper, S. K. & Dean, C. 2012 A year in hypoxia: epibenthic community responses to severe oxygen deficit at a subsea observatory in a coastal inlet. *PLoS One* **7**, e45626. (doi:10.1371/journal.pone.0045626)

5. Matabos, M., Piechaud, N., De Montigny, F., Sarradin, P.-M. & Sarrazin, J. 2015 The VENUS cabled observatory as a method to observe fish behaviour and species assemblages in a hypoxic fjord, Saanich Inlet (British Columbia, Canada). *Can. J. Fish. Aquat. Sci.* **72**, 1–13. (doi:10.1139/cjfas-2013-0611)

6. Cuvelier, D., Legendre, P., Laes, A., Sarradin, P. & Sarrazin, J. 2014 Rhythms and Community Dynamics of a Hydrothermal Tubeworm Assemblage at Main Endeavour Field – A Multidisciplinary Deep-Sea Observatory Approach. *PLoS* **9**, e96924. (doi:10.1371/journal.pone.0096924)

7. Declerck, S. A. J., Coronel, J. S., Legendre, P. & Brendonck, L. 2011 Scale dependency of processes structuring metacommunities of cladocerans in temporary pools of High-Andes wetlands. *Ecography (Cop.).* **34**, 296–305. (doi:10.1111/j.1600-0587.2010.06462.x)

8. Borcard, D., Legendre, P., Avois-Jacquet, C. & Tuomisto, H. 2004 Dissecting the spatial structure of ecological data at multiple scales. *Ecology* **85**, 1826–1832. (doi:10.1890/03-3111)

9. Chu, J. W. F. & Tunnicliffe, V. 2015 Oxygen limitations on marine animal distributions and the collapse of epibenthic community structure during shoaling hypoxia. *Glob. Chang. Biol.* **21**, 2989–3004. (doi:10.1111/gcb.12898)

10. Chu, J. W. F. & Gale, K. S. P. 2017 Ecophysiological limits to aerobic metabolism in hypoxia determine epibenthic distributions and energy sequestration in the northeast Pacific ocean. *Limnol. Oceanogr.* **62**, 59–74. (doi:10.1002/lno.10370)
